# To Go or Stay: The Development, Benefit, and Detriment of Tissue-Resident Memory CD8 T Cells during Central Nervous System Viral Infections

**DOI:** 10.3390/v11090842

**Published:** 2019-09-11

**Authors:** Taryn E. Mockus, Heather M. Ren, Shwetank, Aron E. Lukacher

**Affiliations:** 1 Department of Neurology, Ohio State University Wexner Medical Center, Columbus, OH 43210, USA; 2 Department of Microbiology and Immunology, Pennsylvania State University College of Medicine, Hershey, PA 17033, USA; 3 Biocon Bristol-Myers Squib Research and Development Center, Biocon Park, Bengaluru 560099, India

**Keywords:** CD8 T cells, resident memory T cells, viral infection, central nervous system

## Abstract

CD8 T cells coordinate immune defenses against viral infections of the central nervous system (CNS). Virus-specific CD8 T cells infiltrate the CNS and differentiate into brain-resident memory CD8 T cells (CD8 bT_RM_). CD8 bT_RM_ are characterized by a lack of recirculation and expression of phenotypes and transcriptomes distinct from other CD8 T cell memory subsets. CD8 bT_RM_ have been shown to provide durable, autonomous protection against viral reinfection and the resurgence of latent viral infections. CD8 T cells have also been implicated in the development of neural damage following viral infection, which demonstrates that the infiltration of CD8 T cells into the brain can also be pathogenic. In this review, we will explore the residency and maintenance requirements for CD8 bT_RM_ and discuss their roles in controlling viral infections of the brain.

## 1. Introduction

The brain has long been considered an immune privileged organ. New research that shows that the brain has conventional lymphatic drainage, anatomical niches that harbor resident populations of macrophages and dendritic cells, and glia that operate as innate immune cells, runs counter to this concept [1,2,3,4]. The immune privileged hypothesis of the brain arose, in part, from the unique cells that populate the central nervous system (CNS). Many cells in the brain, such as neurons and mature oligodendrocytes, are postmitotic and terminally differentiated, creating a fragile environment that is extremely sensitive to infection and infiltration. Despite this, many virus families, such as polyomaviruses (e.g., JC polyomavirus (JCPyV)), herpesviruses (e.g., varicella zoster virus (VZV) and herpes simplex virus (HSV)), enteroviruses (e.g., poliovirus), and flaviruses (e.g., Zika virus and West Nile Virus (WNV)) are neurotropic or gliatropic. The consequences of these viral brain infections—e.g., meningitis, myelitis, encephalitis, and demyelination—contribute to a significant health burden worldwide. Immune responses that can control these viral infections must negotiate a trade-off between viral control and immune-mediated damage.

For many viral infections of the brain and other organs, viral control is mediated by CD8 T cells [5]. These cells secrete effector molecules such as granzymes, perforin, and interferon (IFN) γ. IFNγ is implicated in the control of many neurotropic infections, including DNA viruses such as HSV and VZV, RNA viruses such as Sindbis virus (SINV) and measles virus (MV), and parasites such as *Toxoplasma gondii* (*T. gondii*) [6,7,8,9,10]. However, IFNγ is also highly harmful to brain-resident cells. CD8 T cells that respond to infections in non-lymphoid organs can differentiate into tissue-resident memory cells (T_RM_), which remain in the tissue and survey for reinfection [11]. While it is well documented that CD8 T_RM_ are necessary to protect non-lymphoid tissues from reinfection and reactivation of latently infected cells, an understanding the processes that influence the formation and maintenance of CD8 T_RM_ is limited, especially in non-barrier organs such as the brain. This review explores the role of CD8 T cells in the control of viral infection in the brain, with a specific focus on the development, maintenance, and function of virus-specific brain CD8 T_RM_ (CD8 bT_RM_). For clarity, CD8 bT_RM_ refers to resident memory CD8 T cells in the CNS and T_RM_ refers to resident memory CD8 T cells in other, non-CNS organs.

## 2. CD8 T cell Response to Viral Infections of the Brain

Brain-resident innate immune sentinels, such as microglia and astrocytes, are the first responders to viral infection in the CNS [3,12,13]. In addition to their antimicrobial activities, these and other cells secrete chemokines that recruit innate and adaptive immune cells, such as CD8 T cells, into the brain to aid in pathogen clearance or containment [3,12,13]. The activation of CD8 T cells following brain viral infection occurs peripherally in the majority of viral infections. Antigens and antigen-specific effector CD8 T cells have been isolated from peripheral blood following infection from CNS-tropic infections [14,15]. Despite their ubiquitous presence following viral infection, the migration of T cells into the brain might depend on the virus and the type of cells infected. For example, following brain WNV infection, IL-1β produced by infiltrating macrophages was crucial for the recruitment of effector CD8 T cells and their adherence to brain microvasculature endothelial cells, which allowed the T cells to extravasate into the brain parenchyma [16]. Following brain vesicular stomatitis virus (VSV) infection, CD8 T cells were found in clusters, presumably at sites of prior infection, which suggested that virus location dictated the location of VSV-specific effector CD8 T cells [11]. Conversely, immune reconstitution syndrome in patients with progressive multifocal leukoencephalopathy (PML-IRIS), a demyelinating disease caused by JCPyV infection of the brain, was marked by an increase in blood brain barrier (BBB) permeability, suggesting that the extravasation of these JCPyV-specific CD8 T cells across the BBB may not be as strongly dependent on macrophages or virus location [17]. However, during coronavirus encephalitis, T cell entry into the brain was dependent on VCAM-1 expressed by BBB endothelium [18]. Despite robust recruitment of effector CD8 T cells during early, acute infection, there was little evidence that CD8 T cells continue to infiltrate the brain during late stages of persistent infection. In recent studies from our laboratory, we found that CD8 T cells persist in the brain after depletion of circulating CD8 T cells [19]. It is important to recognize that many factors may affect the magnitude, duration, and site of CNS infiltration by virus-specific CD8 T cells. These include differences in viral tropism for CNS-resident cells and their responses to infection, the anatomic location of infectious foci, and interactions with virus-specific and/or bystander T and B cells, as well as with myeloid cells and innate lymphoid cells. Understanding the many complex and dynamic elements regulating CD8 T cell infiltration in the CNS is a major challenge to the viral neuroimmunology field.

**Figure 1:**
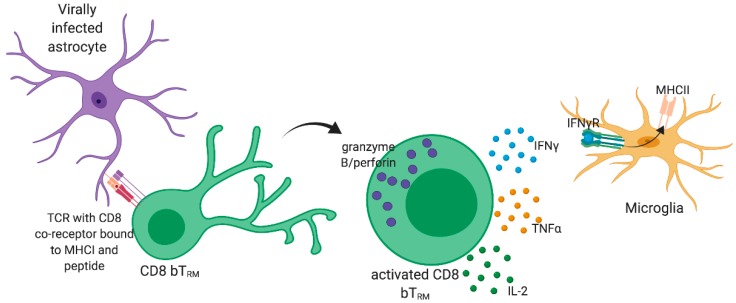
**CD8 T_RM_ have multiple effector functions.** After stimulation by cognate antigen through their T cell receptors TCRs, CD8 T_RM_ may become activated to produce cytotoxic granules such as granzyme B and perforin and/or cytokines such as IFNγ. These granules induce apoptosis in target cells, but this process may require additional help from cytokines. IFNγ is the most common cytokine produced by CD8 T_RM_, but CD8 T_RM_ can also produce other cytokines, such as TNFα and IL-2. IFNγ has many functions in the brain, including increasing MHCII expression of microglia and other antigen presenting cells. The figure was created with BioRender.com.

Once at the site of infection inside the brain, CD8 T cells contribute to virus control. Effector CD8 T cells have multiple functions, as depicted in Figure 1, and these functions are carried out through the production of a number of effector molecules. Effector molecules produced by CD8 T cells fall into two broad categories: cytotoxic granules and cytokines. Cytotoxic granules are modified lysozymes that contain perforin, granzymes, and granulysin. These molecules work synergistically on the target cell to induce apoptosis. Effector CD8 T cells also release cytokines such as tumor necrosis factor α (TNFα), IFNγ, and IL-2. These cytokines can work in concert with the cytotoxic granules to induce apoptosis in the target cell. They also have other roles, such as the activation of macrophages or the induction of MHC I on target cells, which contribute to host defense. The ability of T cells to control neurotropic and gliatropic infections was demonstrated by the failure to clear CNS infections in T cell-deficient mice [20]. However, the brain is largely intolerant of immune activity. Thus, CD8 T cell activity must balance pathogenicity against viral control [21].

IFNγ constitutes a critical regulatory checkpoint in the development and progression of neuroinflammation. IFNγ signaling is thought to reduce viral spread by promoting an antiviral state in infected cells and neighboring uninfected cells (Figure 1) [22]. This potent antiviral activity of IFNγ is critical for the control of many CNS-tropic viral infections, such as MV, HSV, SINV, lymphocytic choriomeningitis virus (LCMV), and mouse hepatitis virus (MHV) [10,23]. Indeed, other common effector mechanisms of CD8 T cells, such as perforin-mediated killing, are not effective during LCMV infection of the CNS, but IFNγ is necessary for viral clearance [23,24]. IFNγ is an important immunoregulator, as shown by unchecked infiltration of neutrophils and fatal encephalitis in HSV-infected mice deficient in IFNγ [25]. However, the role of IFNγ in viral infection remains contentious because IFNγ is known to promote a proinflammatory state in the brain. Endothelial cells of the BBB are susceptible to IFNγ produced by local T cells during neuroinflammation, and IFNγ signaling in these cells increases the permeability of the BBB and the trans-epithelial migration of leukocytes [26,27]. IFNγ also promotes a proinflammatory state in microglia and other infiltrating innate immune cells, which is marked by an increased release of neurotoxic inflammatory cytokines, such as IL-1β and TNFα, and phagocytic activity [28]. During brain mouse polyomavirus (MuPyV) infection, we found that IFNγ produced by virus-specific CD8 T cells in the brain upregulated the expression of MHC class II on microglia, demonstrating that IFNγ from CD8 T cells can directly promote inflammation [29]. Furthermore, IFNγ release from CD8 T cells is toxic to brain resident cells, as shown by the deafferentation of neurons following brain LCMV infection and CD8 T cell infiltration [30]. These studies suggest that IFNγ has simultaneous, conflicting activities during viral infection in the brain: (1) constraining neuropathology by controlling the virus and (2) increasing neuropathology by promoting inflammation.

In addition to promoting neuroinflammation, CD8 T cells and other immune cells can potentiate demyelination of the CNS [31]. Infection of the brain with the JHM strain of MHV (JHMV) results in focal myelin loss, despite viral clearance from the adaptive immune response [32]. Rag1^-/-^ and severe combined immunodeficiency disease (SCID) mice do not develop demyelination during JHMV infection, but do following transfer of JHMV-specific T cells [33]. CD4 and CD8 T cells responding to CNS infection by JHMV are each capable of inducing demyelination [31]. Similarly, the infiltration of T cells during PML-IRIS promotes cell death and demyelination, despite efficient control of virus infection [34]. During chronic Theiler’s murine encephalomyelitis virus (TMEV) infection, the progression of symptoms and demyelination was found to correlate with an increase of virus-specific CD8 T cells, which demonstrated that the adaptive immune response influences demyelination in many different CNS-tropic viral infections [33]. A molecular understanding of the mechanisms of T cell-mediated demyelination are unknown. IFNγ and TNFα can induce cell death in oligodendrocytes during inflammatory conditions, suggesting that these cytokines may cause demyelination [35,36]. In addition to CD8 T cells, inflammatory demyelinating lesions in TMEV, JCPyV-PML IRIS, and JHMV infections are enriched with microglia and macrophages, and it is known that microglia and macrophages are primary contributors to myelin loss [33,34,37]. CD8 T_RM_ secrete numerous chemokines that recruit myeloid cells to sites of viral infection and inflammation [38]. Collectively, these studies indicate that CD8 T cells must thread the needle between causing catastrophic damage and maintaining homeostasis in the CNS.

## 3. Development of CD4 and CD8 T_RM_

The CD8 T cell response to infection can be divided into four phases: (1) priming; (2) contraction; (3) memory; and (4) recall [39]. After priming in lymphoid organs, CD8 T cells rapidly proliferate, acquire an effector function, and migrate to sites of inflammation. About 90–95% of the newly generated CD8 T cells die during the contraction phase, while the remaining 5–10% of virus-specific CD8 T cells differentiate into memory cells that self-renew in the absence of antigens [39]. CD8 T cell memory subsets are defined by their trafficking, localization, and surface marker expression. Memory CD8 T cells are conventionally categorized into three subsets: Central memory T cells (T_CM_), effector memory T cells (T_EM_), and T_RM_ [40]. Activated T cells infiltrate infected and, potentially, uninfected non-lymphoid tissue during the effector phase of the T cell response, then may become permanently established in tissues as T_RM_ [41,42]. The anatomical location of CD8 T_RM_ allows them to protect and rapidly clear reinfection in their tissue of residence. Similar to other non-lymphoid tissues, infection of the brain results in the infiltration and persistence of pathogen-specific CD8 bT_RM_. CD8 bT_RM_ are maintained independently of the circulation and persist in the brain, presumably at sites of prior infection [11]. The resident CD8 T cell population in the brain represents a bona fide CD8 T_RM_ population that provides frontline defense against reinfection by attacking infected cells and communicating with immune cells in the circulation.

Similar to CD8 T cells, CD4 T cells also form T_CM_, T_EM_, and T_RM_ subsets. While there are similarities between CD4 and CD8 T_RM_, clear differences exist between them. One of the most notable differences is that CD4 T_RM_ do not express CD103 (αE integrin) [43]. However, mucosal CD4 T_RM_ express most of the other canonical CD8 T_RM_ phenotypic markers such as CD69 and granzyme B [44]. CD4 T_RM_ are also similar transcriptionally and functionally to CD8 T_RM_ in terms of recall responses [43]. Most studies on CD4 T_RM_ focus on peripheral non-lymphoid tissues, leaving many unanswered questions about CD4 bT_RM_. Similar to Beura et al., we observed little-to-no expression of CD103 by CD4 T cells in the brain (HMR, unpublished data), but CD4 and CD8 bT_RM_ have similar expression levels of CD69 thirty days after MuPyV or VSV brain infections (HMR, unpublished data) [43]. Research on HSV infection in the skin and female reproductive tract suggest that there may be two populations of CD4 T_RM_: a static population that remains parked in the tissue and another dynamic population that migrates between the tissue, draining lymph nodes, and the blood [45]. While the importance of these two populations of CD4 T_RM_ is unknown, the prevalence of either population seems to depend on the virus and its preferred location. For example, during vaginal HSV infection, CD4 T_RM_ are maintained independently of the circulation and are sustained by a network of macrophages, which suggests that reliance on antigen or the tissue microenvironment may affect CD4 T_RM_ retention [46]. Conversely, HSV-specific CD4 T_RM_ in the skin equilibrate with the circulation during steady state and rapidly re-accumulate upon reinfection [47]. These studies suggest that CD4 T_RM_ constitute a heterogeneous population that has some shared and distinct characteristics with CD8 T_RM_.

## 4. Phenotype and Transcriptomes of CD8 T_RM_

CD8 T_RM_ are phenotypically, metabolically, and transcriptionally distinct from other CD8 T cell subsets [48,49,50]. CD8 T_RM_ are marked by their expression of CD69 and CD103, although not all T_RM_ will express these surface molecules to the same level [38]. As shown in Table 1, the relative expression of various surface molecules and transcription factors in and on CD8 bT_RM_ vary among different viral infections. CD8 T_RM_ also adopt a Ly6C^lo^, CD122^lo^, CD127^-/int^, CD62L^lo^, and granzyme B^+^ expression profile, but this can also differ with the tissue and the type of virus infection [49]. T_RM_ share a core gene signature of downregulated tissue-egress genes, such as *Krüppel-like factor 2* (*Klf2*) and *S1pr1* (which encodes a receptor for sphingosine-1 phosphate, S1P1); downregulated cytokine responsive transcription factors, such as the T-box transcription factors T-bet and Eomesodermin (Eomes); and upregulated transcription factors Hobit and Blimp-1 [48]. CD8 T_RM_ from different organs may express some parts of this core signature, but CD8 T_RM_ are heterogeneous and will not express all of the core CD8 T_RM_ simultaneously, supporting the speculation that multiple CD8 T_RM_ subsets may exist [49].

While the factors regulating the phenotypic heterogeneity of CD8 bT_RM_ are only partly known (e.g., TGFβ induction of CD103), there is evidence that the nature of the virus infection guides CD8 bT_RM_ development. For example, only 30–40% of CD8 bT_RM_ become CD103^+^ during persistent MuPyV infection, but close to 90% of CD8 T cells will express CD103 following brain infection with VSV, an acutely resolving infection, which suggested that the chronicity of the viral infection may affect the frequency of CD8 CD103^+^ bT_RM_ [53,55,60]. A comparison of CD103^+^ and CD103^−^ CD8 bT_RM_ to splenic CD103^−^ CD8 T cells revealed increased effector activity and differential regulation of chemokine and cytokine genes, such as CXCL10, CCL3, and S1P1 during *T. gondii* infection. Further, we found that CD103^+^ and CD103^−^ CD8 bT_RM_ are transcriptionally similar [19,55]. Despite their genetic similarities, there is speculation that CD103^+^ and CD103^−^ CD8 bT_RM_ may have different functions. There is some evidence that CD103 dictates the residency of CD8 T cells in particular tissues, as shown by the loss of LCMV-specific CD8 T_RM_ from the intestinal epithelium when CD103 expression was decreased [66]. However, we have found that MuPyV-specific CD103^+^ and CD103^−^ CD8 bT_RM_ are maintained equally in the brain following systemic CD8 T cell depletion, which demonstrated that CD103 expression is not required for maintenance in this infection model [60]. Another hypothesis suggested that CD103 may determine the location of CD8 T_RM_ within the tissue parenchyma because CD103 binds to E-cadherin [67]. This has been shown in other tissues, such as the gut, but no difference was found in the location of CD103^+^ and CD103^−^ CD8 bT_RM_ following infection with *T. gondii* or LCMV [53,68]. This is possibly because the expression of E-cadherin on normal brain cells, such as neurons and oligodendrocytes, is minimal [53,69]. It has also been suggested that CD103 may dictate the degree of motility of CD8 T_RM_, but this has been shown in just a few non-lymphoid tissues [51,53,70]. Using IFNγ-eYFP reporter mice, which allow in situ visualization of IFNγ production, we found that CD103^+^ CD8 bT_RM_ had an increased production of IFNγ compared to CD103^−^ CD8 bT_RM_ following MuPyV intracerebral rechallenge, despite CD103^+^ and CD103^−^ bT_RM_ being equally capable of making IFNγ after ex vivo stimulation with viral peptides [19]. This dichotomy in IFNγ production has also been shown for CD103^+^ and CD103^−^ CD8 T_RM_ subsets in the gut [68]. These results suggest that the CD103^+^ T_RM_ subset is better poised to respond rapidly upon reinfection. However, the mechanisms regarding the differentiation of these two subsets are currently unknown and may reflect differences in proximity to virally infected cells, cytokines exposed to during development, and time of infiltration into the tissue parenchyma.

## 5. PD-1 Expression on CD8 bT_RM_

PD-1 expression is traditionally considered a marker of T cell exhaustion, which is a state of T cell dysfunction characterized by progressive loss of effector function, metabolic abnormalities, and poor responses following infection [71]. Recent work suggests that high PD-1 expression may also enable memory CD8 T cells to survive and retain memory function in the setting of a persistent infection [71]. We found that brain CD8 T cells express PD-1 during MuPyV infection, while memory CD8 T cells in the spleen do not, despite similar viral loads between the two organs during persistent infection [60]. We further found that the expression of PD-1 was independent of viral dose or inflammatory status and that the *Pdcd1* locus was demethylated in brain CD8 T cells, but not splenic CD8 T cells, which suggested that increased PD-1 expression is CD8 T cell-intrinsic [60]. This work and the work of others have shown that high expressions of PD-1 and engagement of the PD-1:PD-L1 pathway promotes CD8 bT_RM_ differentiation and maintenance [29,58,60]. MuPyV and murine cytolomegalovirus (MCMV) brain infections establish a PD-1^+^ CD8 bT_RM_ population (Table 1) [58,60]. During MuPyV infection, high PD-1 expression was correlated with an improved function in CD8 bT_RM_ upon rechallenge with homologous virus [29]. Similarly, the expression of PD-1 and engagement of its ligand, PD-L1, led to improved CD8 bT_RM_ differentiation in mice infected with MCMV, as shown by a reduced frequency of CD69^+^CD103^+^ CD8 T cells in PD-1^-/-^ mice or following PD-L1 blockade [58]. Memory CD8 T cells also expressed high levels of PD-1 in other non-lymphoid tissues during persistent viral infection, demonstrating that PD-1 promotes resident memory differentiation in several non-lymphoid tissues [72].

Recent studies suggest that PD-1 restrains neuroinflammation, in addition to its effects on CD8 bT_RM_ development. We found that MuPyV-specific CD8 T cells expressed more IFNγ when stimulated with viral peptide in the presence of PD-L1^-/-^ bone marrow-derived dendritic cells [29]. Furthermore, nanostring gene expression analysis of the brain microenvironment in PD-L1^-/-^ mice revealed increased expression of neuroinflammatory markers during acute MuPyV infection [29]. Similarly, PD-1^+^ CD8 T cells damaged fewer axons during mouse coronavirus infection compared to their PD-1^lo^ counterparts [73]. The treatment of mice with a PD-L1 fusion protein, which amplified PD-1 function during experimental cerebral malaria, a complication marked by excessive CD8 T cell infiltration into the brain, ameliorated BBB disruption, and reduced CD8 T cell cytotoxicity [74]. The importance of PD-1 in modulating CD8 T cell responses is underscored by clinical data showing that JCPyV-specific CD8 T cells have increased PD-1 expression and blockade of PD-1 on JCPyV-specific CD8 T cells improved T cell responses in patients with PML [75]. The ability of PD-1 expression in CD8 T cells to contain inflammation has been shown in other nonlymphoid tissues. For example, CD8 T_RM_ isolated from the lungs of former smokers also expressed high levels of PD-1 [76]. Together, these results suggested that PD-1 expression on CD8 T_RM_ promotes the maintenance of CD8 T cells, improves viral control, and restrains inflammation, thereby protecting the tissue from extensive damage by both the immune system and virus alike.

Despite its demonstrated role in CD8 T cell memory responses, the mechanisms underlying PD-1-regulated control of CD8 T_RM_ differentiation remain unknown. Glial cells express PD-L1, which increases during inflammatory events and viral infections [77]. We have found that MuPyV-infected glial cells expressed high levels of PD-L1, suggesting that infected glial cells may directly affect the differentiation of CD8 bT_RM_ through engagement of PD-1 [29]. However, no direct interaction between PD-L1^+^ glial cells and PD-1^+^ CD8 T cells has been reported. Thus, additional studies are warranted to clarify the importance and complexity of PD-1 signaling and CD8 bT_RM_ differentiation.

## 6. Factors Influencing the Differentiation of CD8 bT_RM_

**Figure 2:**
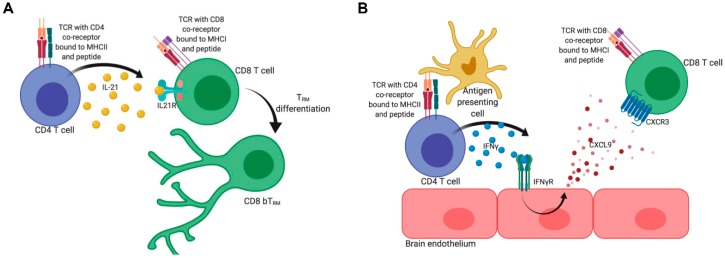
**Potential mechanisms of CD4 T cell help in the brain.** (**A**) When stimulated through their TCR, CD4 T cells produce IL-21. TCR stimulation to the CD8 T cells can upregulate their expression of IL21R. IL-21 from CD4 T cells binds IL21R on CD8 T cells to induce CD8 T_RM_ differentiation. (**B**) CD4 T cells produce IFNγ after TCR stimulation, which binds the IFNγR on brain endothelium to induce CXCL9 production. CXCL9 creates a gradient that CXCR3-expressing CD8 T cells follow to arrive at the site of the infection/inflammation. The figure was created with BioRender.com.

Naïve CD4 and CD8 T cells receive three signals during priming: stimulation of the T cell receptor (TCR) by antigen; costimulation via surface receptors such as CD28; and inflammatory cytokines. It is well-documented that the priming of CD8 T cells also requires the help of CD4 T cells, predominately through CD4 T cell licensing of antigen presenting cells, but recent work has shown that CD4 T cell help is also necessary during the effector and memory phases of CD8 T cell differentiation [78]. The continued importance of CD4 T cell help to CD8 T cell responses is underscored by the incidence of opportunistic infections in HIV/AIDS patients [79]. We recently reported that MuPyV-specific unhelped CD8 T cells (i.e., CD8 T cells primed in the absence of CD4 T cells) had decreased expressions of canonical tissue-resident memory surface markers, a continued dependence on CD8 T cells in the circulation, and a decreased ability to control homologous viral reinfection [19]. Furthermore, acquired CD4 T cell deficiency, modeled by delaying systemic CD4 T cell depletion, also impacted the differentiation of CD8 bT_RM_ and decreased the ability of these cells to control reinfection [19]. Together, these findings reveal an intimate association between CD4 T cells and the homeostasis of functional CD8 bT_RM_ to persistent brain viral infection. Our findings fit with other studies on WNV infection showing that unhelped CD8 T cells do not become CD8 bT_RM_ [54]. The exact mechanism by which CD4 T cells help CD8 T cells become CD8 T_RM_ is unknown. Recent work has identified a few pathways, two of which are highlighted in this review (Figure 2) as potential CD4 T cell-derived mechanisms driving CD8 T_RM_ differentiation.

While many reports have documented the role of CD4 T cell-derived IL-21 in modulating the effector functions of CD8 T cells during acute and chronic viral infections, CD4 T cell-derived IL-21 has only recently been linked to CD8 bT_RM_ cell differentiation [80,81,82,83,84,85]. During early JHMV infection of the brain, CD4 T cells produced IL-21 and CD8 T cells upregulated IL21R [84,86]. IL21R deficiency had no effect on the number of CD8 T cells in the brain during JHMV infection, but CD8 T cells had impaired granzyme B and IFNγ production, which made the normally acute JHMV infection persistent [84,86,87]. Similarly, IL-21^-/-^ mice had fewer IFNγ-producing CD4 and CD8 T cells during brain *T. gondii* infection, which coincided with increased parasite burdens [87]. While not in the context of brain viral infection, IL-21 has been shown to modulate CD103 expression on CD8 T cells in lymphopenic and homeostatic conditions in the small intestine [88]. While the link between IL21R signaling and CD8 bT_RM_ differentiation has not been clearly established, it is worth noting that the effector functions of CD8 T cells, such as granzyme B expression and IFNγ production, have been correlated with the CD8 T_RM_ phenotype, suggesting that the changes in CD8 T cells observed during IL-21 deficiency may be due to alterations in CD8 T_RM_ differentiation (Figure 2A) [19,53,55,60,88].

IFNγ from CD4 T cells has also been shown to modulate CD8 T_RM_ development (Figure 2B). However, unlike IL-21, CD4 T cell-derived IFNγ may act by primarily promoting CD8 T cell entry into the site of infection [89,90]. For example, IFNγ from CD4 T cells stimulated the production of the chemokines CXCL9 and CXCL10, trafficking chemokines that bind to CXCR3, a receptor commonly expressed by effector CD8 T cells, during HSV infection of the female reproductive tract [89]. CXCR3 has been shown to be important for the entry of CD8 T cells into the brain during WNV encephalitis and experimental cerebral malaria [8,91]. Additionally, a loss of IFNγ from CD4 T cells was associated with a reduced frequency of virus-specific CD8 T cells localized to the airway epithelium following influenza infection [90]. The authors further found that CD4 T cell-derived IFNγ was responsible for the upregulation of CD103 on virus-specific lung CD8 T_RM_, suggesting that CD4 T cell-derived IFNγ may also directly affect CD8 T_RM_ differentiation [90]. The efficacy of IL-21 and IFNγ in modulating CD8 T_RM_ differentiation may vary based on the duration of infection and the cells in the surrounding tissue parenchyma. These and other studies highlight that the nature of CD4 T cell help to CD8 T_RM_ development is complex.

The differentiation and maintenance of CD8 T_RM_ may also depend on the tissue microenvironment. For example, memory CD8 T cells favor oxidative phosphorylation for their maintenance. Pan et al. recently demonstrated that CD8 T_RM_ in the skin express the fatty acid-binding proteins Fabp4 and Fabp5, which are required for free fatty acid uptake; deletion of these receptors significantly decreased the number of skin T_RM_, but did not affect other memory CD8 T cells, such as those in the spleen [50]. While circulating memory CD8 T cells also primarily utilize oxidative phosphorylation, CD8 T_CM_ and T_EM_ make their own fatty acids, which suggested that CD8 T_RM_ are uniquely dependent on the tissue microenvironment in which they reside. Tissue microenvironments vary widely, which may affect not only the metabolites available to CD8 T_RM_, but also the maintenance, function, phenotype, and longevity of these cells within the tissue. Indeed, it is well documented that the maintenance requirements for CD8 T_RM_ differ based on the type of virus and the tissue infected [92]. For example, CD8 T cells in the skin after HSV infection and in the salivary gland and kidney after LCMV infection require IL-15 for their maintenance [93,94]. However, IL-15 is not required for the maintenance of LCMV-specific CD8 T_RM_ in other non-lymphoid tissues such as the female reproductive tract and small intestine [94,95]. Likewise, TGFβ is required for the development of CD8 T_RM_ in the skin following HSV infection [93]. Not all CD8 T_RM_ require TGFβ, as shown by the development and maintenance of a TGFβR-deficient CD103^−^ T_RM_ population in the gut following *Yersinia pseudotuberculosis* infection [68]. Cells in the surrounding tissue parenchyma and immune cells can secrete these cytokines, especially TGFβ, suggesting that the local tissue environment and other immune cell subsets affect CD8 T_RM_ differentiation.

The virus infection itself can also dictate the development of CD8 T_RM_. In most non-lymphoid tissues, such as the gut, female reproductive tract, and brain, the differentiation of CD8 T_RM_ occurs in an antigen-independent manner [60,66,70]. However, in the lung, local antigen encounter is required for the differentiation of CD8 T_RM_ [76]. Furthermore, the high and persistent antigen loads found during chronic viral infections promote CD8 T cell exhaustion, which affects the function of CD8 T_RM_ and their ability to survive in persistently infected tissue [78]. The impact of chronic antigen exposure on the stability of CD8 T_RM_ is poorly defined, with most studies focusing on CD8 T_RM_ development following acutely resolving viral infections. Clearly, additional studies are needed to understand the differences in the requirements for differentiation and maintenance of CD8 T_RM_ generated during chronic and acute viral infections.

## 7. Importance of CD8 bT_RM_ in the Control of Virus Reinfection and Viral Latency in the CNS

Studies in parabionts have shown that CD8 T_RM_ mediate the efficient control of reinfection in non-lymphoid tissue, which is even more effective than that mediated by their circulating counterparts [92]. CD8 T_RM_ possess many unique capabilities that allow them to respond rapidly during reinfection. Upon recognition of cognate antigen, CD8 T_RM_ immediately increase IFNγ expression, which recruits circulating antigen-experienced CD8 T cells and other innate immune cells to the tissue [96]. The antigen-experienced CD8 T cells recruited from the periphery do not displace the original CD8 T_RM_, thus ensuring durable protection in tissues prone to reinfection [97,98]. Additionally, CD8 T_RM_ amplify the response of the recruited cells by proliferating in response to antigen exposure, despite having low levels of homeostatic proliferation [97]. Concurrently, the IFNγ released from CD8 T_RM_ induces a broadly active antipathogen response in the entire tissue, enhancing pathogen control [99]. However, although efficient, this alarm function of CD8 T_RM_ is dependent on their ability to detect antigens. It has been shown that CD8 T_RM_ continuously survey the non-lymphoid tissue in which they reside, thereby increasing the probability of encountering a pathogen or an antigen presenting cell [100]. These characteristics of CD8 T_RM_ underscore their role as first responders upon reinfection in non-lymphoid tissue.

Similar to other non-lymphoid tissues, CD8 bT_RM_ are an autonomous barrier against reinfection or resurgence of latent infection. It has been shown that CD8 T cells block HSV-1 reactivation in the ophthalmic branch of the trigeminal nerve, demonstrating a dynamic interaction between CD8 T cells and infected cells [101]. Similarly, CD8 bT_RM_ are essential for protection from reinfection by other CNS-tropic viruses, such as MuPyV, LCMV, and VSV [11,51,62]. Indeed, following depletion of circulating CD8 T cells, CD8 bT_RM_ were able to control LCMV reinfection, which demonstrated that CD8 bT_RM_ can exert viral control independently of the circulation [51]. The importance of CD8 bT_RM_ is underscored by their ability to balance viral control and immunopathology. CD8 bT_RM_-mediated control of LCMV and HSV-1 reinfection exhibited minimal neuropathology, unlike the extensive immunopathology that resulted from the infiltration of circulating memory CD8 T cells from the periphery [24,51,102]. These studies demonstrate that CD8 bT_RM_ are essential for long-term protection against viral infections in the brain.

## 8. Concluding Remarks

CD8 T cells infiltrating the brain during viral infection promote neuroprotection, but may also trigger neurotoxicity. For example, antiviral effector mechanisms deployed by CD8 T cells, such as IFNγ release, are essential for viral control. Yet, IFNγ also increases neuroinflammation and the recruitment of circulating immune cells, which may cause collateral neuropathology. Virus-specific CD8 T cells remain in the brain and often differentiate into CD8 bT_RM_. These virus-specific CD8 bT_RM_ prevent reinfection and check reactivation of latent viral infections in the CNS. However, recent evidence indicating that the development of autoimmune lesions in the brain may be attributed to CD8 bT_RM_ generated from viral infection early in life suggests that CD8 bT_RM_ may also promote pathogenicity [103].

A rapidly accumulating body of evidence supports the concept that the tissue itself directs the differentiation pathway of CD8 T_RM_, leading to context-dependent differences in phenotype, master transcription factor regulators, metabolism, and requirements for maintenance. Superimposed on these tissue-specific effects are those involving viral infections, such as differences between viruses in cell tropism, whether infections are acutely resolved or persistent (and if so, whether persistence is latent or in a chronic infectious state), and variation in innate responses. Thus, there is a pressing need to fill many gaps in our understanding of the delicate balance CD8 bT_RM_ must strike between controlling viral infections while minimizing pathology in the CNS.

## Figures and Tables

**Table 1:** Description and frequency of resident-memory markers on CD8 bT_RM_ during CNS viral infections.

T_RM_ Marker	Function	Frequency of Marker Expression on CD8 bT_RM_ during Brain Infection		
		Acute Infections:	Persistent Infections:	
CD103	Binds to E-cadherin	VSV: ≤90% LCMV: ≤60% MCMV: <40%	MuPyV, *T. Gondii*: 40–60% WNV: <20%	[11,19,51,52,53,54]
CD69	Antagonizes S1PR1 expression	VSV, LCMV: >80% MCMV: <60%	MuPyV, *T. Gondii*: >80% HSV: <40%	[19,51,52,53,55,56,57]
PD-1	Inhibitory receptor, antagonizes TCR engagement	VSV: <1% MCMV: <25% JHMV: 20–45%	MuPyV: >90% *T. Gondii*: 30–50% WNV: 20–30%	[11,58,59,60,61]
CD62L	Lymphocyte-endothelial cell interactions	MCMV: <5%	MuPyV: <5%	[57,62]
Ki67	General marker of cellular proliferation	JHMV: <5%	MuPyV: <20%	[19,63]
Granzyme-B	Mediates apoptosis in target cells	LCMV: <60% VSV: >30% JHMV: >20%	WNV: <20%	[11,16,51,63]
IFNγ (after stimulation)	Pleiotropic cytokine	JHMV: >30% MCMV: >60% LCMV: >50%	MuPyV: <60% WNV: <10%	[16,51,57,60,64,65]

The frequency of CD8 bT_RM_ expressing canonical T_RM_ markers during different virus infections of the brain is shown. Frequencies listed are from CD8 T cells analyzed during days 15 to >30 post infection.
